# Crickets Are Not a Free Lunch: Protein Capture from Scalable Organic Side-Streams via High-Density Populations of *Acheta domesticus*

**DOI:** 10.1371/journal.pone.0118785

**Published:** 2015-04-15

**Authors:** Mark E. Lundy, Michael P. Parrella

**Affiliations:** 1 University of California Division of Agriculture and Natural Resources, Colusa, CA, United States of America; 2 Department of Entomology and Nematology, University of California Davis, Davis, CA, United States of America; IPK, GERMANY

## Abstract

It has been suggested that the ecological impact of crickets as a source of dietary protein is less than conventional forms of livestock due to their comparatively efficient feed conversion and ability to consume organic side-streams. This study measured the biomass output and feed conversion ratios of house crickets (*Acheta domesticus*) reared on diets that varied in quality, ranging from grain-based to highly cellulosic diets. The measurements were made at a much greater population scale and density than any previously reported in the scientific literature. The biomass accumulation was strongly influenced by the quality of the diet (p<0.001), with the nitrogen (N) content, the ratio of N to acid detergent fiber (ADF) content, and the crude fat (CF) content (*y=N/ADF+CF*) explaining most of the variability between feed treatments (p = 0.02; R2 = 0.96). In addition, for populations of crickets that were able to survive to a harvestable size, the feed conversion ratios measured were higher (less efficient) than those reported from studies conducted at smaller scales and lower population densities. Compared to the industrial-scale production of chickens, crickets fed a poultry feed diet showed little improvement in protein conversion efficiency, a key metric in determining the ecological footprint of grain-based livestock protein. Crickets fed the solid filtrate from food waste processed at an industrial scale via enzymatic digestion were able to reach a harvestable size and achieve feed and protein efficiencies similar to that of chickens. However, crickets fed minimally-processed, municipal-scale food waste and diets composed largely of straw experienced >99% mortality without reaching a harvestable size. Therefore, the potential for *A*. *domesticus* to sustainably supplement the global protein supply, beyond what is currently produced via grain-fed chickens, will depend on capturing regionally scalable organic side-streams of relatively high-quality that are not currently being used for livestock production.

## Introduction

Both within the scientific community [1–8] and more broadly [9–12], insect metabolism has been proposed as a potential mechanism for supplementing the protein demands of a growing human population while reducing the environmental impact associated with current systems of livestock production. It has been estimated that livestock production consumes 30% of crops, 8% of freshwater resources, produces as much as 18% of greenhouse gas (GHG) emissions [13], and greatly contributes to the global misallocation of reactive nitrogen (N) [14]. In addition, protein production must double by 2050 due to increases in human population and per capita demand for protein products [13][15]. If this demand is met through livestock production, GHG emissions are predicted to increase 39%, reactive N mobilization will increase 36%, and biomass appropriation will increase 21% by 2050 (relative to year 2000 levels) [15]. Such trends will exacerbate environmental pollution attributable to livestock production [13] and increase the cost of protein relative to today’s prices [16].

Crickets (Orthoptera; Gryllidae) are among the most widely cultivated insect species. They are consumed directly by humans in many regions of the world, and have been raised on an industrial scale in Western countries for decades, primarily as a source of feed for insectivores in captivity (as domestic pets or at zoos) [1]. Because they utilize ambient energy for their metabolic reactions, have shorter generation time, and possess a greater harvestable protein portion than poultry, swine, and beef, crickets have been reported to recover crude protein more efficiently than these protein sources [1][8][17]. In addition, crickets directly emit a fraction of the GHG [18] and directly consume orders of magnitude less water than some vertebrate livestock [19][20].

Nevertheless, as with most livestock production, commercial cricket production relies on grain products as a feed source. Because the environmental impacts associated with livestock production are in large part a function of the resources required to produce the grain products that poultry, swine and beef consume[13][19][21], if cultivated on a grain-based diet, the environmental benefits of cultivating crickets as an alternative protein source will be mostly defined by their improved feed conversion efficiency for grain products.

However, it has been suggested that crickets might also be successfully cultivated on organic side-streams [17]. Roughly one third of the food produced for human consumption, 1.3 billion tons per year, is lost or wasted [22]. Of that, 222 million tons are consumer generated waste in industrialized countries [22]. To illustrate the magnitude of the potential protein embodied in this waste stream: if the estimated 2.2 million (dry) tons of food waste produced annually in the state of California [23] were recovered as protein via insect metabolism at the rates reported by Collavo et al. [17], approximately 210,000 tons of dietary protein would remain directly in the food system. Protein-capture benefits aside, GHG emissions that result from food waste decomposition in landfills [24][25] could be drastically reduced if waste streams were diverted via insect metabolism.

In addition to the data reported by Collavo et al. [17], other investigators have reported similarly efficient conversion of feed to protein by a variety of insect species [6][8][17][26–28]. However, the contexts in which these results were measured do not generally reflect the scale or the degree of control likely to resemble an economically viable production process. In addition, population densities in these studies were generally low, suggesting that, while the conversion efficiencies reported may be accurate on an individual basis, they may or may not apply to production environments with high-density insect populations. Validating and/or revising feed and protein conversion efficiencies for populations of crickets reared at an economically relevant production scale and density is an essential step to accurately assessing their potential to serve as an alternative source of dietary protein. Further, determining the feed and protein conversion efficiencies of scalable, organic side-streams is a necessary step to determining the potential for crickets to be used as a protein recovery/recycling pathway, especially in protein-poor regions of the world.

Therefore, the objectives of this study were to: 1) measure the biomass and protein capture from feed substrates that varied in digestibility and N content at a production scale of economic relevance using high-density populations of the common house cricket (*Acheta domesticus* (L.) [Orthoptera: Gryllidae]); 2) develop a quantitative metric to explain the relationship between feed quality and cricket biomass gain; and 3) compare feed and protein conversion efficiencies of crickets fed a variety of diets to those of conventional livestock.

## Methods

In a controlled-environment greenhouse located at the University of California, Davis, 15 experimental units were organized in randomized complete blocks with 5 treatments and 3 replications per treatment. From 14 d after hatching until they were either harvested or experienced complete mortality, *A*. *domesticus* populations were administered the following 5 feed treatments ad libitum: 1) a 5:1 ratio of non-medicated poultry starter feed and rice bran (Poultry Feed-PF); 2) the solid, pasteurized, post-process filtrate from a proprietary, aerobic enzymatic digestion process [29] that converts grocery store food waste into 90% liquid fertilizer and 10% solids (the portion used in the experiment) (Food Waste 1-FW1); 3) minimally-processed, post-consumer food waste collected from municipalities throughout California’s Bay Area. This is an abundant, heterogeneous substrate that is typically collected and composted at an industrial scale [30] (Food Waste 2-FW2); 4) a 1:1 ratio of wheat and maize silage prepared at an industrial scale as dairy cow rations and containing approximately 50% non-grain aboveground biomass (straw) (Crop Residue 1-CR1); and 5) a 2:1:1 ratio of poultry manure, wheat straw and rice straw silage prepared according to the principals in Oji et al. [31] (Crop Residue 2-CR2).

The experimental units were modified gaylord shipping boxes, which have the footprint of standard international shipping pallets [1.2m (L) x 1.0m (W) x 0.61m (H)]. The interior of each enclosure was lined with a 4mm clear plastic liner and covered with 122cm x 137cm of nylon mosquito netting to serve as a physical barrier to entrance or exit. To prevent cannibalism and stress-related mortality, 96 egg cartons, 30cm x 30cm in size, were placed on-edge around the periphery of each box. This provided approximately 172800cm^2^ of crawl-able surface area. Access to water was provided by 2 quart-sized poultry water dispensers with cotton and gravel inserted in the dispensing basin to prevent the drowning of newly hatched nymphs. Sides of the water dispensers were sanded to provide purchase for the crickets to crawl vertically. Misting tips with check valves to prevent dripping were affixed at the top interior of each enclosure. To maintain acceptable humidity and provide a dispersed, alternative water source for the large population of crickets, these tips provided pulses of water aimed at the center of the enclosure at automated intervals. In keeping with Clifford et al. [20], temperature (T) and relative humidity (RH) within the greenhouse were maintained at 29.0 ± 2.1 standard deviation (SD) °C and 67.2 ± 14.7 SD %, respectively, over the course of the experiment. Light was provided 24h as in Nakagaki et al. [8].

An egg substrate from Timberline Fisheries [32] consisting of approximately 50,000 *A*. *domesticus* eggs with a hatch rate of 70% was placed into each of the enclosures. The egg substrate was maintained between 80–90% humidity until eclosion. Once eclosion was observed, the substrate was misted twice daily until the nymphs had fully emerged.

Populations in each experimental unit were fed an equivalent diet ad libitum for two weeks post-eclosion. This consisted of the PF1 treatment plus 5% active dry yeast on a dry matter (DM) basis, as suggested by Patton [33]. Absolute biomass gain is small during this early developmental period, yet growth rates in the early developmental stages can affect growth rates throughout the life cycle [34][35]. As such, delaying the initiation of the feed substrate treatments until 14 d after hatching ensured that all populations had equivalent metabolic capacity entering the linear portion of their growth phase. At 14 d after emergence, the 5 feed treatments described above, with a composition detailed in Table 1, were introduced.

**Table 1:** Composition and biomass of feed treatments.

	Treatment					
	PF	FW1	FW2	FW2_end_	CR1	CR2
**Total input biomass, dry weight (g)**	15027	6109	6270	---	1447	608
	± 961	± 272	± 66		± 38	± 1
**%N**	4.0	4.6	2.2	2.2	1.5	1.4
**%ADF**	5.1	24.7	41.6	60.1	25.3	50.1
**%CF**	9.5	19.3	15.8	2.5	2.8	1.1
**%Ash**	9.2	7.2	21.4	28.6	6.4	32.8

Dry weight ± standard error (g) and percent composition of nitrogen (N), acid detergent fiber (ADF), crude fat (CF) and ash in the feed inputs of the various experimental treatments prior to their introduction to the *A*. *domesticus* populations. Also included is the percent composition for the same constituents measured at the end of the experiment in the FW2 treatment. See [36] for explanation of terms.

Fresh weights of all input feed substrates were recorded at the time of feeding. A representative subsample of each substrate was then dried at 60°C until reaching a constant weight to determine its moisture content (MC). The same subsample was then analyzed for constituents that are commonly used to assess feed quality for livestock nutrition [36]. These included total N (N), crude protein content, acid detergent fiber content (ADF), crude fat content (CF), and ash content, which were determined according to AOAC Official Methods 972.43, 990.03, 973.18, 2003.05, and 942.05, respectively [37]. The same measurements were made on the unconsumed biomass of the FW2 treatment at the end of the experiment.

Population growth was monitored every 3 to 4 days by counting and weighing a random sample of 70 individuals from each experimental unit. Because population biomass and mortality are correlated and affected by feed quality [17], the treatments were not harvested simultaneously. Rather, treatment-specific harvests were timed to maximize the total protein output per population based on observed changes in the relationship between biomass per individual over time and estimates of population density. This was accomplished by counting subsets of populations on a unit area basis. For the treatments in which a harvest of the entire population was possible (PF and FW1), all the crickets were extracted from the experimental unit and weighed on a wet weight basis. Subsequently, DM and protein content were determined, as described above, for a representative subsample (5% of wet weight) of the harvested population. Feed conversion ratios (FCR) were calculated as the proportion of DM fed to fresh weight harvested. Protein conversion efficiency (PCE) was calculated as the protein content measured in the harvested biomass, expressed as a percentage of the protein in the input feed substrate.

The relationship between feed quality and biomass gain was analyzed for data recorded 30 d after hatching. This date was chosen for analysis because it represented the latest measurement interval at which a majority of the treatments were still demonstrating positive biomass gains, thereby minimizing potentially confounding effects such as cannibalism. Linear models were fit to the data using the “lm” package in R [38]. First, a significant treatment effect was confirmed via ANOVA. Subsequently, models containing biomass per individual (as the response variable) and feed substrate N, ADF, CF, ash content, and their interactions (as independent variables) were fit iteratively to determine the most parsimonious model. Mean square error, F-values, p-values, and R2-values were determined for the final model via ANOVA.

## Results and Discussion

Concentrations of N, ADF, CF and ash varied widely across the feed treatments (Table 1). The poultry feed treatment (PF), which was composed primarily of maize and soy grain products, was similar in composition to diets fed to populations of *A*. *domesticus* by Nakagaki et al. [8] and Patton [39] and served as the control. It contained only 5.1% ADF, which was between 10 and 21% of the ADF concentrations in the other feed treatments. In addition, the PF treatment had among the highest N (and, therefore, crude protein: AOAC 990.03 [37]) concentration, with 182%, 266% and 286% of the N concentrations in the FW2, CR1, and CR2 treatments, respectively. The post-process filtrate from the enzymatic digestion of food waste (FW1) contained a slightly higher N concentration than the PF treatment (15%) but also contained a 484% higher concentration of ADF. The unprocessed, municipal-scale food waste (FW2) contained among the highest concentrations of ADF, CF and ash and substantially less N than the PF and FW1 treatments (Table 1). The CR1 and CR2 treatments, which were each comprised of approximately 50% non-grain, aboveground biomass from grain crops (straw), contained similarly low relative concentrations of N and CF (Table 1). What differentiated these treatments from each other was the higher ADF and ash contents in CR2 than CR1 (198% and 513%, respectively), resulting from the inclusion of poultry manure in the ensiling process.

**Fig 1:**
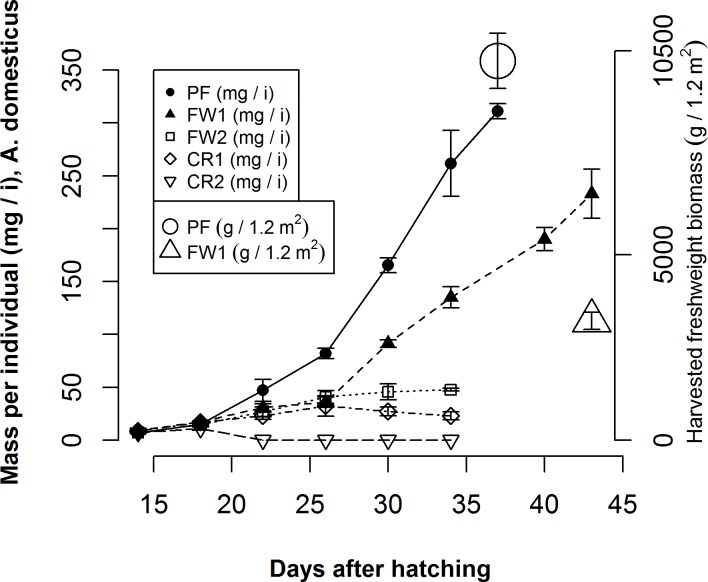
Biomass response of *A*. *domesticus* to variation in diet. Biomass accumulation (mg i^-1^) over time for populations of *A*. *domesticus* receiving Poultry Feed (PF), Food Waste 1 (FW1), Food Waste 2 (FW2), Crop Residue 1 (CR1), or Crop Residue 2 (CR2) diets (left hand axis), and harvested, fresh weight biomass (g / 1.2 m^2^) for PF and FW1 treatments (right hand axis). Vertical lines represent the standard error of the mean between the three replications per treatment.

Cricket growth rates, biomass accumulation (p<0.001), and population viability were strongly determined by the food substrate composition. Populations fed the PF treatment, experienced a 4574% gain in biomass (fresh weight, with moisture content (MC) = 71.9 ± 0.4 standard error (SE) %) per individual between d 14, when the feed treatments were administered, and d 37, when crickets in this treatment were harvested (Fig. 1). This represents a growth rate of 13.2 ± 0.3 SE mg i^-1^ d^-1^ over the treatment period and an overall growth rate of 8.4 ± 0.2 SE mg i^-1^ d^-1^, which is in close agreement with Patton [39] for crickets fed a similar diet. Total population biomass for the crickets in the PF treatment was 10235 ± 746 SE g (Fig. 1), or 8529 g m^2–1^ and 13982 g m^3–1^. Populations fed the FW1 treatment also demonstrated steady biomass gain and grew to a harvestable size. Individuals in this treatment experienced a 2583% gain in biomass between d 14 and d 43 (Fig. 1), representing growth rates of 7.7 ± 0.8 SE mg i^-1^ d^-1^ for the treatment period and 5.4 ± 0.5 SE mg i^-1^ d^-1^ overall. The final population biomass in the FW1 treatment was 3217 ± 233 SE g (Fig. 1), or 2681 g m^2–1^ and 4395 g m^3–1^. None of the populations fed the FW2, CR1 or CR2 treatments survived to a harvestable size. From d 14 until d 26, individuals in the FW1, FW2 and CR1 treatments had similar biomass gains (Fig. 1). However by d 30, biomass per individual was declining in the CR1 treatment and was unchanged in the FW2 treatment, whereas individuals in the FW1 treatment had nearly tripled in size (Fig. 1). Further, estimates of population density were negative for the CR1 treatment by d 22 and by d 30 for the FW2 treatment, and these populations each experienced >99% mortality by d 40 and d 43, respectively. Populations fed the CR2 treatment demonstrated very little growth and had experienced >99% mortality by d 22 (Fig. 1).

The change in constituent concentration of the municipal-scale food waste treatment (FW2) between the initiation and conclusion of the experiment gives an indication about which fractions of this substrate were digestible to the populations in this treatment. The crude fat concentration decreased from 15.8% to 2.5%, while the N content remained unchanged, and the ADF and ash concentrations increased from 41.6% to 60.1% and from 21.4% to 28.6%, respectively. Therefore, it seems probable that the populations in the FW2 treatment were able to survive for a limited period and achieve some biomass gain from the lipids present, but that the protein in the substrate was largely inaccessible, and the fibers and ash were indigestible. Although substances such as marrow from bone fragments, which have a high N concentration, were observed to be completely consumed, they made up such a small proportion of the overall biomass that this change was neither measureable nor sufficient to support the protein requirements of the population. This observation, combined with the unchanged N concentration from the beginning to the end of the experimental period, indicates that the populations in the FW2 treatment were likely protein-starved.

**Fig 2:**
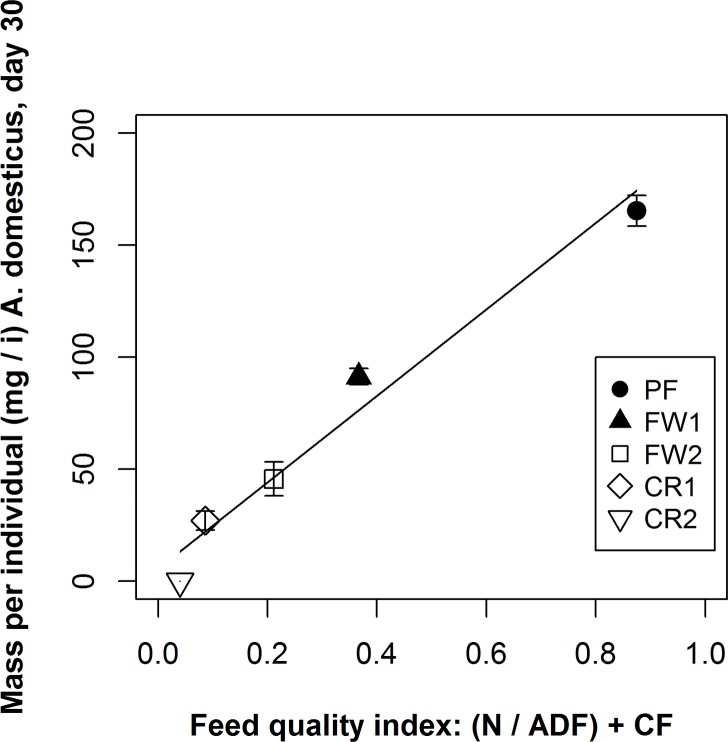
The effect of feed quality on the biomass response of *A*. *domesticus*. Depicts the relationship between feed quality and the biomass response of *A*. *domesticus* populations at 30 days after hatching, where feed quality is characterized by the proportion of nitrogen (N) to acid detergent fiber content (ADF) plus the proportional crude fat content (CF) [y = (N/ADF) + CF] in the feed substrate treatments (p = 0.02; R^2^ = 0.96). Vertical lines represent the standard error of the mean between the three replications per treatment. For ANOVA see Table 2. See [36] for explanation of terms.

As indicated by analysis of the biomass response at d 30 as a function of the feed substrate constituents (Fig. 2; Table 2), the N concentration of the substrate was the primary determinant of biomass gain. On its own, the N content explained 68% of the variation across treatments (p < 0.001), and the ratio of N to ADF explained another 28% of the overall treatment variability (p < 0.001) (Table 2). The CF content explained a small (4%) but significant (p < 0.01) portion of the overall treatment effects (Table 2). The ash content did not significantly affect the biomass response between treatments. However, this may be due to that fact that the feed substrates for the various treatments were fed ad libitum. If a substrate were more limiting to the population as a whole, it is possible that the ash content could factor more prominently. The most parsimonious model to predict biomass response from the range of constituents measured in the feed treatments is:

y=[N/ADF]+CF

where N = proportional N content, ADF = proportional ADF content, and CF = proportional CF content, significant at p = 0.02 with an R^2^ = 0.96 (Fig. 2; Table 2). Because N, ADF and CF concentrations are commonly reported metrics for a variety of organic substrates, the feed quality model reported here may be useful in determining the potential of a particular organic side-stream to serve as a feed source for crickets. However, further work on a wider array of substrates is needed to validate this conclusion, particularly in the feed quality range between 0.37 (FW1) and 0.86 (PF). For example, Ramos-Elorduy et al. [28] fed yellow mealworms (*Tenebrio molitor* (L.) [Coleoptera: Tenebrionidae]) 5 diets that would have had a feed quality range of 0.35–1.09 using the index reported here. However, in that study there was not a clear, linear biomass response as food quality increased. Yet, differences in species and experimental conditions probably factor into this discrepancy. In that regard, the feed quality index reported here should be viewed as a preliminary model.

**Table 2:** ANOVA for the regression depicted in Fig. 2.

	MSE	F-value	p-value
**intercept**	---	---	0.02
**N**	33708	232	<0.001
**N / ADF**	13689	94	<0.001
**CF**	2215	15	<0.01
**residuals**	146	---	—-

The mg i^-1^ of *A*. *domesticus* at 30 days after hatching is explained (p = 0.02; R^2^ = 0.96) by the ratio of nitrogen (N) to acid detergent fiber content (ADF) plus the proportional crude fat content (CF), [(N/ADF) + CF], in the feed substrate treatments. See [36] for explanation of terms.

The feed conversion ratio (FCR) for the populations fed poultry feed (PF) was 1.47, when using the entire fresh-weight cricket biomass as the output, and 1.84 under the assumption, reported by Nakagaki et al. [8], that only 80% of the harvested cricket biomass is directly consumable (Table 3). If the unconsumed feed that remained after harvest were subtracted from the total input biomass, the FCRs would improve to 1.34 and 1.68 for 100% and 80% of the harvested cricket biomass, respectively. These FCRs are higher (less efficient) than the 1.09 and 1.36 average FCRs (at 100% and 80% harvested biomass, respectively) reported by Nakagaki et al. [8] for *A*. *domesticus* populations fed four diets, one of which was a poultry starter diet. However, in addition to differences in the constitution of feed substrates, the population size and density were not comparable between the two experiments. Nakagaki et al. [8] developed FCR estimates from populations of 10 individuals at a density of 1 individual per 14.4cm^2^. In our study, the population in the PF treatment was approximately 32,900 individuals with a density of 1 individual per 5.25 cm^2^. Although this density exceeds the minimum 2.5cm^2^ per individual reported by Patton [39], it is far more crowded than the populations measured by Nakagaki et al. [8]. In addition, when rearing populations in a large-scale environment, it is likely that the structure of the environment will result in greater metabolic costs per individual (e.g., average distance required to procure food and water is greater). Finally, although the mean temperature (29.0 ± 2.1 SD °C) and % relative humidity (67.2 ± 14.7 SD) align with reported optimums for *A*. *domesticus*, the variability of these factors at the greenhouse scale is likely to have been higher than within a laboratory setting, where previous work was conducted. Thus, greater metabolic costs may have been incurred [40].

**Table 3:** Comparative efficiencies of crickets and selected livestock.

	*A. domesticus*		Carp^1^	Chicken	Pork^1^	Beef^1^
	PF	FW1				
**Feed conversion (kg dry feed / kg live weight)**	1.3	1.8	1.5	1.7^2^–2.3^1^	5.9	12.7
**Feed conversion (kg dry feed / kg edible weight)**	1.7^3^	2.3^3^	2.3	2.4^4^–4.2^1^	10.7	31.7
**Protein content (% of edible weight)**	16	16	18	20^1^	14	15
**Protein conversion efficiency (%)**	35	23	30	33^4^–25^1^	13	5

Feed conversion ratios, protein content, and protein conversion efficiency for *A*. *domesticus* in the Poultry Feed-PF and Food Waste 1-FW1 treatments as well as for carp, chicken, pork and beef.

1. Smil [42].

2. Feddes et al. [43].

3. Using 80% edible portion, as in Nakagaki et al. [8].

4. Using 70% dressing percentage (harvestable portion) [44] instead of 55% used by Smil [42] and the feed conversion reported by Feddes et al. [43].

For the FW1 treatment, the FCR was 1.91 for 100% and 2.39 for 80% of the harvested biomass (Table 3). If only the consumed portions of the FW1 treatment were considered, the FCRs would be reduced to 1.82 and 2.28 for 100% and 80% of harvested biomass. These FCRs are slightly higher than those reported by Collavo et al. [17] (1.69 for 100% and 2.12 for 80% of harvested biomass) for *A*. *domesticus* populations fed a “human refuse diet” that consisted of fruit and vegetables peels (34%), rice and pasta (27%), pork and beef meat (11%), bread (11%), cheese skins (11%) and yolk (6%). However, the feed quality and the population size and density differed in these cases as well. The MC (71.9 ± 0.4 SD %) and protein content (15.8 ± 0.6 SD %) did not differ between the PF and FW1 treatments and were also in agreement with the range of estimates for these parameters reported in Collavo et al. [17] and Nakagaki, et al. [8].

It is clear that the biomass response of cricket populations depends, in large part, on the quality of their diet, which has important implications for the sustainability of crickets as an alternative source of protein. The ecological costs of grain-based protein production systems are largely embedded in the production of the grains themselves. For example, Oonincx et al. [18] reported that direct greenhouse gas emission from crickets, mealworms, and other insects were much lower than from swine and beef. Yet, as a proportion of the global warming potential (GWP) from the entire system of production, direct emissions from mealworms only totaled 0.29%, whereas the mixed grain and carrot diet they consumed accounted for 56% of the total [4]. Similarly, the GWP [41] and water footprint [19] for livestock production are largely determined by the quantity of feed consumed. Therefore, the potential for crickets and other insects to be a less resource-intensive form of dietary protein than conventional livestock will depend, in large part, on their improved protein conversion efficiency (PCE) when fed a grain-based diet.

Crickets fed a grain-based diet resulted in a PCE of 35% (Table 3), which is slightly better than the more efficient end of the range reported by other investigators [42–44] for broiler chickens fed a similar diet (Table 3). While there is a theoretical potential for PCE gains by producing crickets on grain-based diets when compared to broilers at the less efficient end of the PCE range reported in Table 3, it should also be noted that mature systems of production, distribution and consumption are already in place for poultry production. These existing efficiencies as well as the reduced energy requirement for rearing poultry might help to explain the discrepancy between the 1.1 kg CO_2_-eq associated with the production of 1 kg of poultry [41] (the low end of a range extending to 5.5 kg CO2-eq / kg [45]) versus the 2.7 kg CO_2-_eq / kg reported by Oonincx et al. [4] for mealworms with similar FCRs and energy requirements as crickets. Even if global demand for crickets were to exist at a much greater scale than it does at present, a novel protein source with little or no PCE improvement compared to chicken is unlikely to justify the investments required to produce crickets at a scale of global significance. Further, the same global forces driving the recent and projected increases in conventional livestock prices [16] will also increase the cost of crickets fed these same diets.

Although the crickets fed the FW1 diet were less productive per unit time and unit area (Fig. 1) and had lower overall PCE than crickets fed the PF diet (Table 3), they may offer greater sustainability gains than crickets fed grains. In addition to avoiding the ecological costs embodied in the grain itself, the conversion of an organic side-stream to dietary protein prevents its deposition in environments with substantial nutrient loss pathways such as landfills and large-scale composting operations [24][25][46]. Yet, whether the FCR and PCE reported for the FW1 diet are sufficient to create an economically viable production system is an important question that is not analyzed here. However, because the quality of the organic side-stream factored largely into whether the crickets could grow successfully, the implication is that higher quality side-streams would be preferred for the production of crickets. As noted by Elferink et al. [47], scalable organic side-streams are already being used in the production of pork, and a scalable substrate that is high in protein content might share demand with swine and other forms of livestock. Therefore, identifying regionally scalable waste substrates of sufficient quality to produce crickets that have no direct competition from existing protein production systems might be the most promising path for producing crickets economically, with minimal ecological impact, and at a scale of relevance to the global protein supply.

## Conclusion

Although it has been suggested that crickets reared for human or livestock consumption may result in a more sustainable supply of protein, this study finds that such conclusions will depend, in large part, on what the crickets are fed and which systems of livestock production they are compared to. When fed grain-based diets at a scale of economic relevance, populations of crickets in this study showed little improvement in PCE compared to broiler chickens fed similar diets. When fed processed, organic side-streams of relatively high quality, cricket populations achieved a harvestable size. Yet, whether crickets could be raised economically on substrates of similar quality and level of processing requires further analysis. The unprocessed and lower-quality organic side-streams tested in this study could not support adequate growth and survival of cricket populations. Therefore, the potential for crickets to supplement the global supply of dietary protein appears to be more limited than has been recently suggested. However, the feed quality index reported here may be useful in identifying regionally specific organic side-streams with the potential to support the scalable cultivation of crickets.

It should be noted that crickets are but one of multiple insect species with potential for augmenting the global supply of protein by capturing organic side-streams and/or converting feed to protein more efficiently than conventional livestock [2][3]. It is possible that other species, such as black soldier fly (*Hermetia illucens* (L.) [Diptera: Stratiomyidae]) are better suited to the bioconversion of low-quality organic side-streams to dietary protein [1][6]. Nevertheless, champions of cultivating insects as a sustainable form of protein should recognize that the efficiency of any insect production system and, therefore, its protein contribution and ecological impact, will depend on the quality of the insect diet.

In order for insect cultivation to sustainably augment the global supply of protein, more work is needed to identify species and design processes that capture protein from scalable, low-value organic side-streams, which are not currently consumed by conventional livestock.
